# Efficacy and safety of antimicrobial de-escalation of treatment for sepsis: A protocol for systematic review and meta-analysis

**DOI:** 10.1097/MD.0000000000023385

**Published:** 2020-12-04

**Authors:** Hong Zhu, Pai Peng, Rui Zhao, Kai-Yu Fang, Shi-Quan Han

**Affiliations:** a Medical Intensive Care Unit; b Ultrasound Imaging Department, Dalian Municipal Central Hospital, DaLian, Liaoning, China.

**Keywords:** de-escalation, efficacy, safety, sepsis, systematic review

## Abstract

**Background:**

Sepsis has become a global healthcare problem and continues to be one of the leading causes of death due to infection. In essence, early recognition and diagnosis of sepsis is needed to inhibit the transition into septic shock, which is correlated with higher mortality. Many studies have suggested antimicrobial de-escalation as one of the strategies to replace the empirical broad-spectrum antimicrobial treatment using a narrower antimicrobial therapy, especially among patients with sepsis. However, antimicrobial de-escalation therapeutic effects in sepsis remains unclear. We therefore performed the present study in an attempt to assess efficacy and safety of antimicrobial de-escalation therapy in patients with sepsis.

**Methods:**

We will carry out a systematic literature search to establish the potentially eligible trials from electronic databases, including EMBASE (1980 to October 16, 2020), MEDLINE via PubMed (1966 to October 16, 2020), Web of Science (1965 to October 16, 2020), Cochrane Library (CENTRAL; 2020, Issue 10), WanFang databases (last searched October 16, 2020), and China National Knowledge Infrastructure (CNKI; last searched October 16, 2020). For this study, the language restrictions are English or Chinese. Two authors independently examined quality based on the Cochrane Risk of Bias Tool V.2.0 and extracted data. Data obtained from the study will be synthesised using applicable statistical methods.

**Results:**

The results of the present study will systematically assess efficacy and safety of antimicrobial de-escalation therapy among patients with sepsis.

**Conclusion:**

The results of the present study will help to establish the efficacy and safety of antimicrobial de-escalation to treat patients with sepsis. It can also help to identify the most efficient and safe therapeutically-relevant method.

**Ethics and dissemination:**

The present study is a meta-analysis and the pooled results are based on published evidence. Therefore, ethics approval is not necessary.

**OSF registration number:**

October 22, 2020.osf.io/93wym. (https://osf.io/93wym/).

## 1 Introduction

Sepsis is considered a multifaceted disorder which advances as a dysregulated host response in regards to diseases. It is related to acute organ dysfunction and is a high-risk condition that can lead to death. In particular, the condition requires urgency in treating, thus it is critical to create awareness of the presenting characteristics. Sepsis impacts over 30 million adults worldwide.^[1]^ Additionally, the incidences of sepsis have significantly increased in the recent 4 decades, and this condition has remained one of the leading causes of deaths worldwide.^[2,3]^ In essence, septic shock is considered a subclass of sepsis, wherein there is a circulatory and metabolic dysfunction.^[4]^ Meanwhile, sepsis is regarded as a substantial burden on society today and has continually affected the older adults disproportionally.^[5,6]^

It is critical to consider the management and of sepsis and septic shock as urgent medical conditions. First, patients screening to identify whether they have signs and symptoms of sepsis seems to facilitate earlier recognition and intervention.^[7,8]^ More importantly, effective treatment needs to emphasise on timely intervention, such as removing the source of the disease. Despite therapeutic innovations, the mortality rate in septic shock is still high.^[9,10]^ In particular, the main causes of death among these patients include refractory multi-organ failure and hypotension. Similar in septic shock, early commencement of treatment is critical as a delay often results in multiple organ dysfunction.^[11]^ At the same time, raid antimicrobial de-escalation therapy can enable clinicians to have a more comfortable feeling regarding considerations that can foster quick administering of broad-spectrum antibiotics instantly subsequent to identifying patients with sepsis. There have been no trials tried to address these challenges related to the suitability of such treatments. Only few studies have evaluated the efficacy and safety of antimicrobial de-escalation to treat patients with sepsis. Moreover, these studies deduce conflicting results. Therefore, we carried out the present study to explore the efficacy and safety of antimicrobial de-escalation therapy in patients suffering from sepsis to establish a new evidence for the control and prevention of sepsis.

## 2 Methods

### 2.1 Registration

The current study was expressed on the Open Science Framework (OSF, http://osf.io/), registration DOI number is 10.17605/OSF.IO/93WYM. This protocol has been drafted under the Preferred Reporting Items for Systematic Review and Meta-Analyses Protocols (PRISMA-P) statement guidelines.

### 2.2 Criteria for considering studies for this review

#### 2.2.1 Types of studies

We will incorporate a randomised controlled trials (RCTs).

#### 2.2.2 Types of participants

Further, we anticipate to incorporate studies on patients of different ages, suffering from sepsis as a result of any micro-organism.

#### 2.2.3 Types of interventions

The experimental intervention will be on any antimicrobial de-escalation therapy for the treatment of sepsis. The control group will obtain a placebo, no intervention, and other intervention method.

#### 2.2.4 Types of outcome measures

##### 2.2.4.1 Major outcomes

The major anticipated outcomes include

1. short-term mortality: mortality estimated at day 28;
2. short-term mortality: mortality estimated at time periods greater than 28 days;
3. all-cause mortality at hospital discharge or after the follow-up period.

##### 2.2.4.2 Minor outcomes

The minor anticipated outcomes include

1. length of intensive care unit stay;
2. length of hospital stay among survivors;
3. individual antimicrobial resistance;
4. re-infection;
5. adverse events.

### 2.3 Search methods for identification of studies

#### 2.3.1 Electronic searches

Further, we intend to carry out a systematic review and search of literature to determine potentially appropriate experiments from electronic databases, such as EMBASE (1980 to October 16, 2020), MEDLINE via PubMed (1966 to October 16, 2020), Web of Science (1965 to October 16, 2020), Cochrane Library (CENTRAL; 2020, Issue 10), WanFang databases (last searched October 16, 2020), and China National Knowledge Infrastructure (CNKI; last searched October 16, 2020). Moreover, language limitations are English or Chinese. Further, we will utilize following MESH terms, free text, and synonyms to explore the above-mentioned databases: sepsis∗, “antimicrobial therapy”, “de-escalation” together with specialized filters for RCTs.

#### 2.3.2 Searching other resources

Here, we intend to search other resources and citations of published references to establish more studies. Also, we intend to get in touch with authors to get supplementary published or un-published data.

### 2.4 Data collection and analysis

#### 2.4.1 Selection of studies

**Figure 1:**
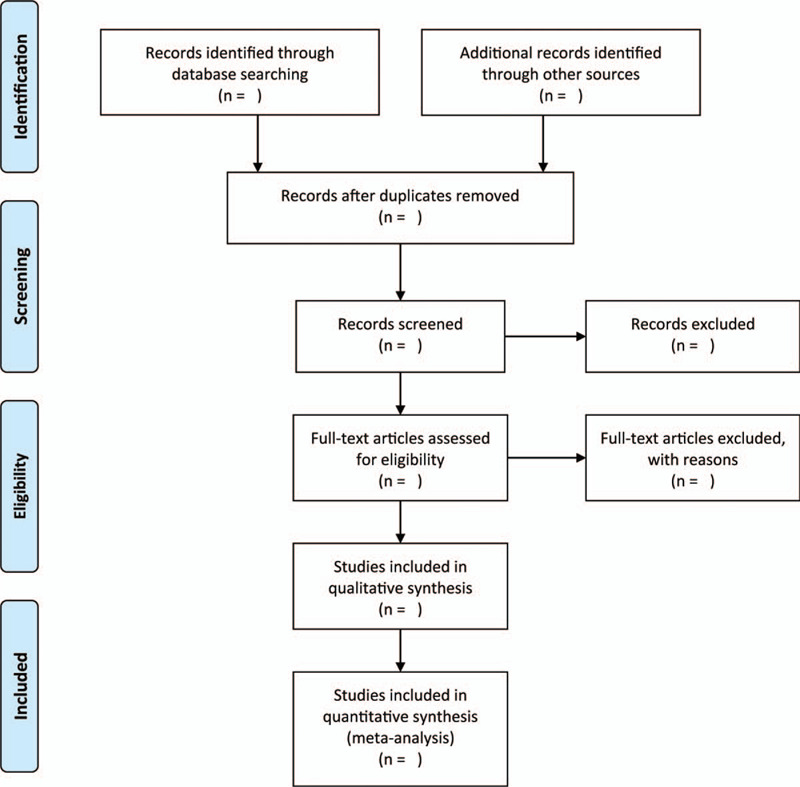
Flow diagram of the literature search.

We will select 2 impartial authors to study the results of the selected trials and independently screen the titles, abstract, and full-text to find out which trials need further evaluation. We will ensure that any difference in opinion are resolved through discussion or by consulting a party a third author. The literature screening process is depicted in Figure 1.

#### 2.4.2 Data extraction and management

The 2 impartial authors will plan to individually obtain data from 5 domains of each included study by using a data extraction form:

1. Study characteristics
  Publication year, duration of the study, language of the study, exact their location, withdrawals
2. Participants
  N, mean age, age range, sex, ethnicity, socioeconomic status, diagnostic criteria, inclusion, and exclusion criteria.
3. Interventions
  Intervention, comparison, other co-interventions, and excluded medications.
4. Outcomes
  Major and minor outcomes stated, collected, and time points reported.
5. Notes
  Trial results as well as the obvious conflict of interest must reveal of trial authors.

Any disagreement in opinion will be addressed through discussion or by consulting an impartial author.

#### 2.4.3 Assessment of risk of bias in included studies

The bias risk identified in the study will be examined impartially by the 2 authors using the Cochrane Collaborations “Risk of bias” tool.^[12]^ The evaluated study features will include:

1. concealment of allocation;
2. outcome assessment;
3. co-interventions;
4. losses to follow up;
5. intention to treat.

Also, we seek to further rank every prospective source of bias as “low risk”, “high risk”, or “unclear risk”. Additionally, we will address differences or disputes between ourselves through discussion, or by involving a third author.

#### 2.4.4 Measures of treatment effect

For the dichotomous variables, we intend to set out to estimate the risk ratio (RR) with 95% confidence intervals (CI), or alternatively Peto odds ratio (OR) with 95% CI if rare events. However, for continuous variables, we will strive to calculate the mean difference (MD) with 95% CI or the standardized mean difference (SMD) with 95% CI.

#### 2.4.5 Assessment of heterogeneity

We will evaluate the heterogeneity using by the *Chi*^*2*^ statistic and *I*^2^ test. If the *P* value is less than .1 or *I*^2^ test more than 50%, there is a significant heterogeneity. We will employ a random-effects model to pool the data^[13]^; otherwise, the fixed-effects model will be used to pool the data.^[14]^

#### 2.4.6 Assessment of reporting biases

We will apply the funnel plots to examine any publication bias if at least 10 trials are available.

#### 2.4.7 Data synthesis

Where it is possible to use meta-analysis, we will pool outcomes using the fixed-effects model as a default. We will apply a fixed-effects model unless there is significant statistical heterogeneity, in which case we will apply the random-effects model.

#### 2.4.8 Sensitivity analysis

Where there are adequate data, we will utilize sensitivity to examine the robustness of our findings by excluding studies with low-quality or unclear methodological data.

## 3 Discussion

Overall, sepsis is a typical disease related to the high mortalities and long-term morbidities, especially among patients who survive. Increasing awareness of the condition and the ongoing campaigns to improve quality care to better understand the evidence-based methods to managing the problem, which has led to the advanced outcomes. Likewise, empirical broad-spectrum antimicrobial treatment seeks to achieve sufficient antimicrobial therapy and reduce mortality. Still, the risk that empirical broad-spectrum antimicrobial treatment might endanger patients to the overuse of antimicrobials. At the same time, antimicrobial de-escalation is regarded as an approach that might substitute the empirical broad-spectrum antimicrobial treatment when a narrower antimicrobial therapy is employed. Still, studies that assess the efficacy and safety of antimicrobial de-escalation to treat patients with sepsis are limited, while the results are also controversial. Therefore, this study will systematically assess the efficacy and safety of antimicrobial de-escalation therapy in patients with sepsis using past and existing clinical studies to establish a basis for clinical diagnosis and treatment.

## Author contributions

**Conceptualization:** Hong Zhu, Shi-Quan Han.

**Data curation:** Hong Zhu.

**Formal analysis:** Hong Zhu.

**Funding acquisition:** Hong Zhu.

**Investigation:** Pai Peng.

**Methodology:** Pai Peng.

**Project administration:** Pai Peng, Rui Zhao, Kai-Yu Fang.

**Resources:** Pai Peng, Rui Zhao, Kai-Yu Fang.

**Software:** Rui Zhao.

**Validation:** Kai-Yu Fang.

**Visualization:** Shi-Quan Han.

**Writing – original draft:** Shi-Quan Han.

**Writing – review & editing:** Shi-Quan Han.
